# Cutaneous manifestations of monoclonal gammopathy

**DOI:** 10.1038/s41408-022-00661-1

**Published:** 2022-04-11

**Authors:** Jean-Sebastien Claveau, David A. Wetter, Shaji Kumar

**Affiliations:** 1 Division of Hematology, Mayo Clinic Rochester, Mayo Clinic, MN USA; 2 Department of Dermatology, Mayo Clinic, Rochester, MN USA

**Keywords:** Myeloma, Diseases

## Abstract

Monoclonal gammopathy associated with dermatological manifestations are a well-recognized complication. These skin disorders can be associated with infiltration and proliferation of a malignant plasma cells or by a deposition of the monoclonal immunoglobulin in a nonmalignant monoclonal gammopathy. These disorders include POEMS syndrome, light chain amyloidosis, Schnitzler syndrome, scleromyxedema and TEMPI syndrome. This article provides a review of clinical manifestations, diagnostics criteria, natural evolution, pathogenesis, and treatment of these cutaneous manifestations.

## Introduction

The monoclonal gammopathies are a spectrum of disorders characterized by clonal proliferation of plasma cells or lymphoid cells resulting in secretion of a monoclonal protein with clinical manifestations ranging from asymptomatic to serious and even life-threatening disease. This secreted monoclonal protein (M-protein) is typically an intact immunoglobulin but can also be present as light chains unbound to any heavy chain (free light chain, FLC) and can be detected in the blood and/or urine [1].

The clone may remain indolent over a prolonged period, as in monoclonal gammopathy of undetermined significance (MGUS). MGUS is asymptomatic and consistently precedes the development of smoldering myeloma and multiple myeloma. Multiple myeloma represents the most common symptomatic and typically fatal part of the disease spectrum, with increasing tumor burden resulting in a multitude of clinical features like hypercalcemia, renal insufficiency, anemia, and bone disease (CRAB). However, the toxicity of secreted M-protein, alteration of the host immune system, secretion of cytokines, and plasma cell infiltration can produce severe manifestations even with a very small and quiescent clone. The kidney, peripheral nerves, and skin are the principal organs that can be involved [2]. Thus, monoclonal gammopathy associated with dermatological manifestations, grouped along with monoclonal gammopathy of clinical significance (MGCS) is a well-recognized complication. According to Daoud & al., monoclonal gammopathy of skin significance can be divided into four different groups (Table 1) [3]. In Group I, infiltration, extension, and proliferation of malignant plasma cells are associated with cutaneous manifestations. In Group II, a nonmalignant monoclonal gammopathy is strongly associated with cutaneous disease. This can be caused by deposition of all or part of the monoclonal immunoglobulin, autoantibody activity, cytokine-mediated, and by an unknown mechanism. Group III are miscellaneous cutaneous manifestations anecdotally associated with monoclonal gammopathy. Small studies or case series should have clearly shown the correlation between Group III cutaneous manifestations and monoclonal gammopathy. Group IV are cutaneous conditions, symptoms, and complications related to M proteins, but not specific for monoclonal gammopathy. This may include adverse reactions to therapy used for the treatment of plasma cell disorders. Hyperviscosity-related gum bleeding in Waldenström macroglobulinemia (WM) and cutaneous infection associated with immunodeficiency are other examples of Group IV cutaneous conditions. Group III and IV cutaneous manifestations will not be discussed further in this review.

**Table 1:** Monoclonal gammopathies and associated skin diseases.

Group I • Waldenström macroglobulinemia • Systemic immunoglobulin light chain amyloidosis • Cryoglobulinemia • Plasmacytoma • Osteosclerotic myeloma (POEMS syndrome)
Group II • High association ◦ Scleromyxedema ◦ Scleredema ◦ Necrobiotic xanthogranuloma ◦ Plane xanthoma ◦ Schnitzler syndrome • Low association ◦ Pyoderma gangrenosum ◦ Sweet syndrome ◦ Leukocytoclastic vasculitis ◦ Neutrophilic dermatosis • Unknown ◦ Erythema elevatum diutinum ◦ Subcorneal pustular dermatosis
Group III • Miscellaneous cutaneous disorders described in association with monoclonal gammopathies
Group IV • Miscellaneous cutaneous signs or symptoms including purpura, pruritis, infection, adverse reactions to medications, etc.

Adapted from ref. ^3^.

**Fig. 1:**
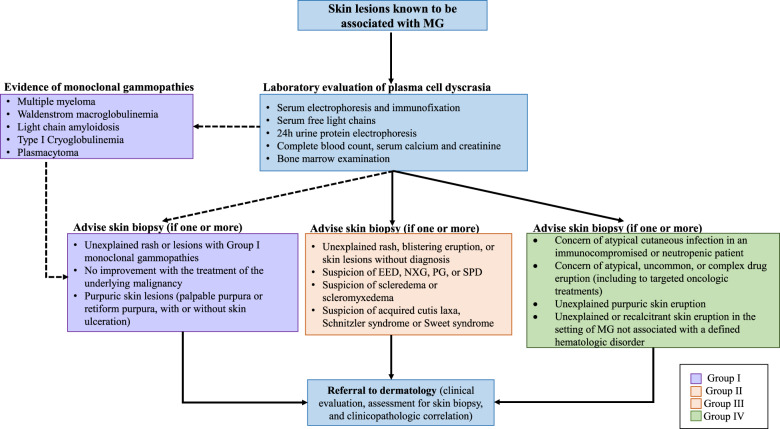
Management algorithm of cutaneous manifestations associated with monoclonal gammopathy. Abbreviations: EED erythema elevatum diutinum, MG monoclonal gammopathy, NXG necrobiotic xanthogranuloma, PG pyoderma gangrenosum, SPD subcorneal pustular dermatosis.

Investigation of unexplained cutaneous lesions should be carried out by a hematologist and dermatologist working together. Rheumatology, internal medicine, and ophthalmology consultation may also be relevant in certain situations. As described in the management algorithm (Fig. 1), every patient with a monoclonal gammopathy (or MGUS) with new unexplained cutaneous lesions should be investigated. In these cases, we recommend skin biopsy and bone marrow examination in addition of the standard laboratory evaluation of plasma cell dyscrasia. Additionally, patients with chronic unexplained cutaneous lesions associated with a monoclonal gammopathy should be investigated. In patients with plasma cell malignancies, investigations should be considered if cutaneous lesions are persistent despite treatment of the underlying condition. Finally, in patients with worsening or recalcitrant skin eruptions (particularly in the Group II category) that do not respond to skin-directed therapy, treatment of the underlying monoclonal gammopathy/malignancy could be considered as a primary treatment for the skin condition (since the skin condition may be “reactive” to the underlying monoclonal gammopathy). In this article, we summarize the clinical manifestations, diagnosis criteria, histopathological finding, and management of these uncommon cutaneous manifestations.

## Group I

### POEMS

POEMS syndrome is a rare monoclonal plasma cell disorder and refers to polyradiculoneuropathy (P), organomegaly (O), endocrinopathy (E), monoclonal gammopathy (M), and skin changes (S). Additional features include sclerotic bone lesions, Castleman disease, papilledema, pleural effusion, ascites, erythrocytosis, and thrombocytosis [4]. The diagnostic criteria for POEMS are shown in Table 2. The heavy chains in POEMS syndrome can be IgA, IgG, or more rarely IgM. However, the light chain is almost always lambda [5]. In patients without serum or urine M-protein (less than 12% of cases), a monoclonal lambda plasma cell is usually found [6]. There is a Castleman variant POEMS syndrome that does not have a monoclonal gammopathy [4].

**Table 2:** POEMS syndrome diagnostic criteria.

Mandatory major criteria (both required)	• Polyneuropathy • Monoclonal plasma cell-proliferation disorder
Other major criteria (at least one required)	• Castleman’s disease • Sclerotic bone lesions • Vascular endothelial growth factor elevation
Minor criteria (at least one required)	• Organomegaly (splenomegaly, hepatomegaly, or lymphadenopathy) • Extravascular volume overload (edema, pleural effusion, or ascites) • Endocrinopathy (adrenal, thyroid, pituitary, gonadal, parathyroid) • Skin changes • Papilledema • Thrombocytosis
Other symptoms and signs	Clubbing, weight loss, pulmonary hypertension, restrictive pulmonary syndrome, diarrhea, thrombotic disease, hyperhidrosis

Both mandatory major criteria must be fulfilled in addition to at least 1 other major criteria and 1 minor criteria. Adapted from ref. ^4^.

The skin lesions associated with POEMS syndrome include hyperpigmentation, acrocyanosis, plethora, telangiectasia, hypertrichosis, and skin thickening [7]. Glomeruloid hemangioma, red-purple lesions on the trunk and proximal extremities are also present in many patients [8]. Patients can exhibit Raynaud phenomenon, flushing, and white nails. Sclerodermoid change characterized by skin thickening, facial lipoatrophy [9], and calciphylaxis may also occur [7, 10, 11].

Some studies have suggested a causative relationship between VEGF and skin lesions. Pihan & al. clearly demonstrated that raised VEGF seems to correlate with a high sensitivity and specificity in the diagnosis of POEMS syndrome [12]. Indeed, elevated VEGF may lead to hypertrichosis by stimulating local vascularization and hyperpigmentation by increasing melasma lesions [13, 14]. Stromal and vascular change present in digital clubbing may be explained by VEGF when released with platelet-derived growth factor (PDGF) on platelet aggregation [15]. Upregulation of vascular endothelial growth factor receptor (VEGFR-1) by VEGF seems to play a central role in the development of hemangiomas [16].

In patients with 1 or 2 bone lesions and no bone marrow involvement, radiation therapy is the treatment of choice. Radiation therapy in limited disease can be curative and even improve cutaneous lesions [17]. If there are more than 2 bone lesions and/or a clonal plasma cell on bone marrow biopsy, systemic therapy can be considered despite no randomized trial available. Lenalidomide-based therapy followed by autologous stem cell transplantation is associated with a favorable prognosis [18–20].

### Waldenström macroglobulinemia cutis

WM is defined as a clonal proliferation of lymphoplasmatic cells producing a monoclonal IgM protein. WM can be associated with small, pearly flesh-colored papules on the extensor surfaces of the extremities. These lesions are asymptomatic and may appear before or at the diagnosis of the WM. On histopathology, there are IgM deposits in the dermis without associated amyloid or cellular infiltration. Specimens are periodic acid-Schiff positive and show hyaline amorphous and granular eosinophilic deposits involving the upper and mid dermis [21].

Several cases of WM with bullous involvement have been reported [22, 23]. The typical clinical manifestations are blisters, erosions, or papules on the dorsum of the hands. The location of the vesicles was generally subepidermal with dermal deposits of IgM [24]. Some authors hypothesized that the pathogenesis can be similar to epidermolysis bullosa acquisita or bullous systemic lupus erythematous [25]. In a case report, a patient with hyperkeratotic colored papules achieved complete response with rituximab while the second rapidly progressed and died of disease progression [26]. Another patient with subepidermal bullae was successfully treated with rituximab, cyclophosphamide, and prednisone [27].

### Light chain amyloidosis (AL)

Immunoglobulin light chain amyloidosis (AL) is due to an aberrant production of monoclonal kappa or lambda light chain that forms the substrate for amyloid fibril formation, and its deposition in different organs leading to organ damage and consequent clinical manifestations [28].

**Fig. 2:**
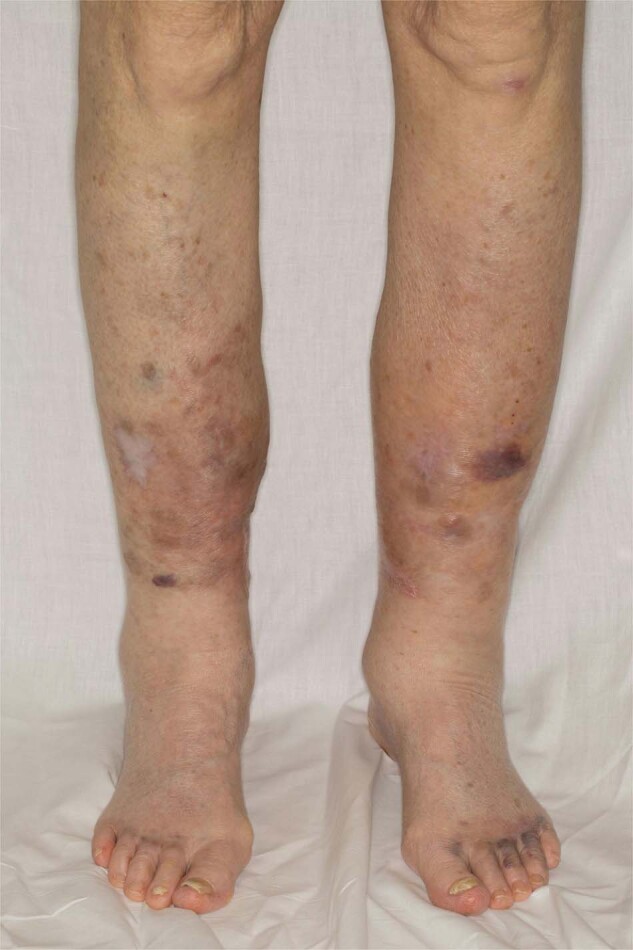
Light chain amyloidosis. Pink-red to purpuric plaques and nodules involving the lower extremities.

The dermatological manifestations in AL can be myriad (Fig. 2). Periorbital and facial purpura are classical signs of AL amyloidosis [29]. Petechia, scattered non-traumatic ecchymoses, nodules, alopecia, and scleroderma-like changes of the skin are seen. Papules with dome-shaped, waxy, translucent appearances are common. Oral involvement includes macroglossia, induration of the tongue, and gum bleeding. A case of uvula AL amyloidosis has also been reported [30]. Purpura may be the result of amyloid infiltration of blood vessels, acquired factor X deficiency, and increased fibrinolysis [31–33].

Nail dystrophy characterized by onychorrhexis, onychoschizia, and nail splitting has been reported. The distal nail plate involvement is generally more important than the proximal nail. Nail dystrophy may be misdiagnosed as trachyonychia and a careful evaluation of nail lesions must be done [34].

The diagnosis of amyloidosis is based on histopathological examination of tissues. Electron microscopy or Congo red may confirm the presence of amyloid deposits [35]. Skin biopsy usually demonstrates faintly eosinophilic amorphous masses of amyloid deposits in the dermis and subcutaneous tissues [3]. Mass spectrometry is the gold standard to confirm the amyloid protein composition and to distinguish AL amyloidosis from other forms [36–38].

In low-risk patients, autologous stem cell transplantation with high-dose melphalan is recommended with or without bortezomib or daratumumab-based induction [39–42]. Bortezomib consolidation should also be considered [43]. In intermediate-risk and high-risk patients, daratumumab in combination with cyclophosphamide, bortezomib, and dexamethasone (DARA-CyBorD) should be the first option [44, 45].

### Cryoglobulinemia

Cryoglobulinemia is a disorder characterized by the presence of cryoglobulins which precipitate at temperatures below 37 degrees Celsius. Cryoglobulinemia has been classified into three different types. The type 1 cryoglobulinemia occurs in monoclonal gammopathies including MGUS, multiple myeloma, WM and chronic lymphocytic leukemia [46, 47].

In type II cryoglobulinemia, the cryoglobulins are a mixture of monoclonal immunoglobulin (typically IgM or otherwise IgG or IgA) in combination with rheumatoid factor (RF) and polyclonal IgG. Type II cryoglobulins are associated with hepatitis C infection, systemic lupus erythematous, Sjogren’s syndrome, or less frequently infection (hepatitis B virus or HIV) [48, 49]. Type III cryoglobulins have exclusively polyclonal IgG and polyclonal IgM with RF activities. They are associated with autoimmune disease and infections, mainly hepatitis C virus [50].

**Fig. 3:**
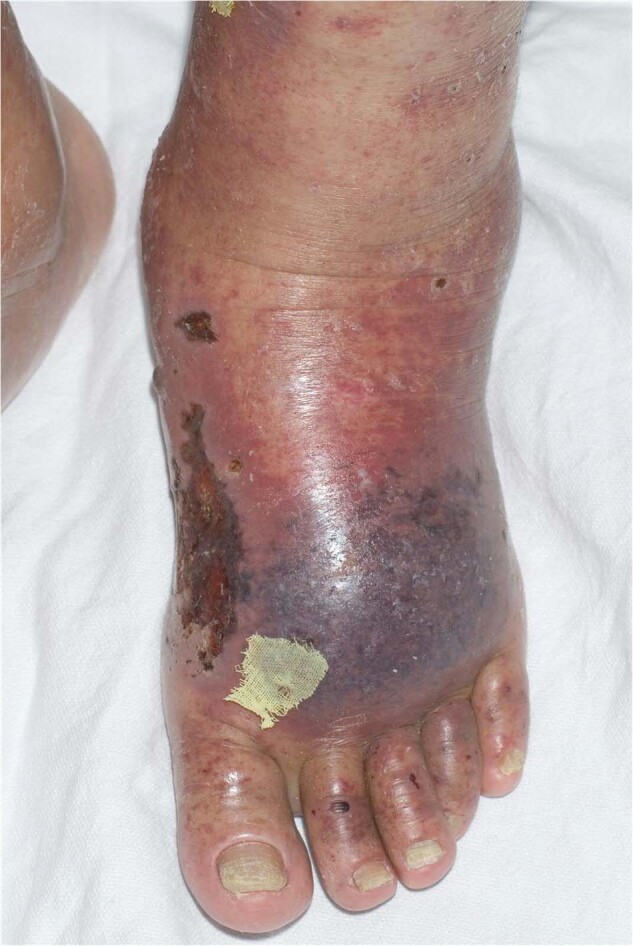
Type I Cryoglobulinemia. Purpura and stellate ulcerations of the foot.

Cohen et al. published a description of cutaneous lesions found in 72 patients with cryoglobulinemia. Many patients had inflammatory macules or papules, hemorrhagic crusts, scarring, acrocyanosis, and livedo reticularis (Fig. 3). Hyperpigmentation of the leg after repeated episodes of purpura is frequent [51]. Ulcers and infarctions were also much more frequent in type I cryoglobulinemia than in type II and III [52]. Monoclonal cryoglobulins with high thermal insolubility may cause more severe ulceration [53]. The histopathological finding includes erythrocyte extravasation, hyaline thrombosis, non-inflammatory sequelae, and rarely vasculitis features. Type I cryoglobulinemia classically presents with non-inflammatory retiform (net-like) purpura with microscopic changes of microvascular occlusion; while types II and III cryoglobulinemia typically present with palpable purpura and microscopic changes of leukocytoclastic vasculitis [54]. The treatment should be directed against the underlying plasma cell disorder. In patients with acute kidney injury, bortezomib is the first choice of treatment for type 1 cryoglobulinemia, while in patients with neuropathy lenalidomide may be considered. If there is hyperviscosity, rituximab introduction should be delayed and plasmapheresis should be used [55]. In mixed cryoglobulinemia, treatment should include antiviral therapy with or without rituximab and corticosteroids according to the severity of the disease [56, 57].

### Plasmacytoma

Plasmacytoma is defined as a neoplastic proliferation of plasma cells and can involve skin. Skin plasmacytoma may be characterized by one or more skin lesions and can be associated with monoclonal gammopathy. This can take three different forms: (1) in association with multiple myeloma, (2) without association with multiple myeloma, and (3) direct extension from an underlying bone lesion. WM, heavy-light chain disease can develop skin plasmacytoma [58, 59]. IgD multiple myeloma would also present an increased risk for skin plasmacytoma in comparison with other isotypes [60].

**Fig. 4:**
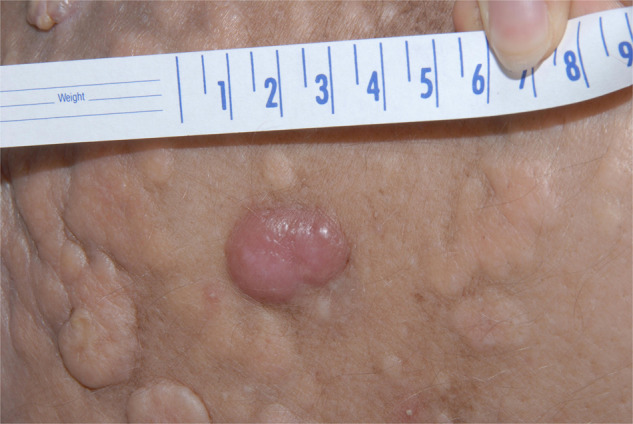
Cutaneous plasmacytoma. Solitary pink-erythematous dermal nodule.

Patients with skin plasmacytoma usually present with red, violaceous, non-tender nodules and occasionally with diffuse erythematous rash (Fig. 4). An erythematous patch overlying a solitary bone plasmacytoma and associated with local adenopathy (AESOP syndrome) has also been described [61]. Histopathological studies demonstrate clonal infiltration by plasma cells [51, 62, 63]. Current treatment options include localized radiation therapy, local surgery, and systemic treatment according to the number of tumors and their characteristics [64], but typically would require systemic therapy when part of myeloma.

## Group II

### Schnitzler syndrome

Schnitzler syndrome is a chronic urticaria associated with monoclonal gammopathy. This late onset acquired autoinflammatory disease was described by Liliane Schnitzler in 1972 [65]. Typically, an IgM gammopathy is found (mainly kappa). However, some cases of IgG are also reported (variant Schnitzler syndrome). The pathogenesis of Schnitzler syndrome is poorly understood and the role of the paraprotein is unclear. NLRP3-related autoinflammatory disease (NLRP3-AID), previously called Cryopyrin-associated periodic syndromes are due to a gain-of-function mutation in NLRP3. Patients with NLRP3 presented similar clinical manifestations to patients with Schnitzler syndrome and both conditions show good response to IL-1 inhibition [66]. However, no study has reported a NLRP3 mutation in patients with Schnitzler syndrome [67, 68]. This mutation, therefore, does not seem to play a role in Schnitzler pathogenesis. Other studies found an increase of IL-1 beta and IL-6 by peripheral blood mononuclear cells [69, 70]. However, the mechanism of these chemokine upregulations remains unclear. High CCL2 levels have been found in Schnitzler patients and may be important in the pathogenesis of the disease [71]. More studies are needed to elucidate the pathogenesis of Schnitzler syndrome.

The urticarial rash (which may be non-pruritic) is described by rose or red macules or gently raised papules. The rash can be associated with dermographism and is mostly present on the trunk and extremities. Skin biopsy demonstrates neutrophilic perivascular and interstitial inflammation. Fibrinoid necrosis of vessels, endothelial swelling, and dermal hemorrhage are generally not present [72]. The term “neutrophilic urticarial dermatosis” has been used to describe the characteristic cutaneous clinical and histopathologic findings of Schnitzler syndrome, although similar findings can be seen in other systemic diseases such as systemic lupus erythematosus, adult-onset Still disease, and periodic fever syndromes [72, 73] Many patients present with joint pain, bone pain, myalgia, and asthenia. The Strasbourg criteria must be fulfilled to confirm the diagnosis (Table 3) [74].

**Table 3:** Schnitzler syndrome classification diagnostic criteria.

Mandatory major criteria	• Chronic urticarial rash • Presence of monoclonal IgM or IgG gammopathy
Minor criteria	• Recurrent fever • Abnormal bone remodeling with or without pain • Neutrophilic dermatosis on skin biopsy • Leukocytosis and/or elevated C-reactive protein (CRP)
Definite diagnostic	• Two mandatory major criteria and at least 2 minor criteria if IgM and at least 3 minor criteria if IgG
Probable diagnostic	• Two mandatory major criteria and at least 1 minor criteria if IgM and at least 2 minor criteria if IgG

Adapted from ref. ^74^.

The treatment of choice for Schnitzler syndrome is IL-1 inhibition with anakinra, canakinumab, or rilonacept. Several studies show that anakinra relieves symptoms in only a few hours after administration [75–78]. A phase II randomized placebo-controlled trial demonstrated that canakinumab, an IL-1 beta monoclonal antibody, significantly reduced clinical manifestations and inflammatory markers [79]. Rilonacept, in a prospective trial, was also effective to induce a rapid clinical response and reduced inflammation [80]. However, IL-1 inhibition does not reduce M-protein and has no impact on the natural history of the disease.

### Necrobiotic Xanthogranuloma

Necrobiotic xanthogranuloma (NXG) is a non-Langerhans histiocytosis characterized by firm yellow or red-orange papules, plaques, and nodules. NXG is strongly associated with a monoclonal gammopathy of type IgG-kappa [81]. No M-proteins are found in 9–19% of cases with NXG. Some cases of IgA or IgG-lambda have been reported [82, 83]. Periorbital skin lesions are the most common site of involvement, but it can occur on the trunk and extremities in the absence of facial lesions [84]. Ocular changes include proptosis, blepharoptosis, scleritis, enlargement of lacrimal glands, and restricted ocular mobility [85]. Atrophy, telangiectasia, ulceration, and violaceous borders are reported. Elevated erythrocyte sedimentation rate (ESR) and leukopenia are common findings. Histopathological findings include granulomatous inflammation in the dermis extending into subcutaneous fat. The granulomas are alternating with foci of collagen necrobiosis and are associated with epithelioid and foamy histiocytes in addition to lymphocytes, plasma cells, and multinucleated giant cells, also called *Touton cells* [86].

NXG is a chronic disease characterized by the progression of existing lesions if not treated. There is no existing randomized clinical trial considering its low prevalence. Alkylating agents chlorambucil and melphalan are the first line agents and retrospective studies have suggested their efficacy with or without corticosteroids [86–88]. High-dose dexamethasone and intravenous immunoglobulin can also be beneficial for NXG [89–93]. Lenalidomide, dapsone, and plasmapheresis are reserved for refractory cases [94–96].

### Plane xanthoma

Diffuse normolipemic plane xanthoma associated with monoclonal gammopathy is characterized by xanthelasma (periorbital plane xanthoma) and diffuse plane xanthoma of the head, neck, trunk, shoulders, or extremities. Xanthomas normally occur in hyperlipidemic patients, however, in rare cases it can occur in patients with normal lipid profiles. Normolipemic plane xanthoma can be associated with MGUS, multiple myeloma, acute myeloid leukemia, lymphoma, and Castleman disease. The skin lesions are described as yellowish-orange plaques [97]. The pathogenesis is unknown, but immune complex formation between antibodies and lipoproteins seems to cause accumulation of lipids in macrophages [98]. Plane xanthoma should be differentiated from necrobiotic xanthogranuloma by their diffuse and plane patches. Necrobiotic xanthogranuloma are more polymorphic and tend to be described as red-brown, violaceous, or yellowish cutaneous plaques, papules, or nodules [99]. In patients with limited lesions, surgical resection or ablative laser therapy can be done [97, 100]. Otherwise, systemic treatment with bortezomib, melphalan, and/or high-dose corticosteroids can be done to achieve hematologic and cutaneous remission [98, 99].

### Scleromyxedema

**Fig. 5:**
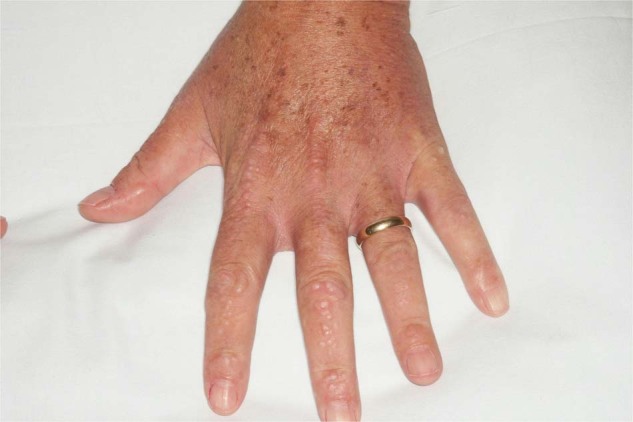
Scleromyxedema. Firm, waxy, dome-shaped papules of the dorsal fingers and hand.

**Fig. 6:**
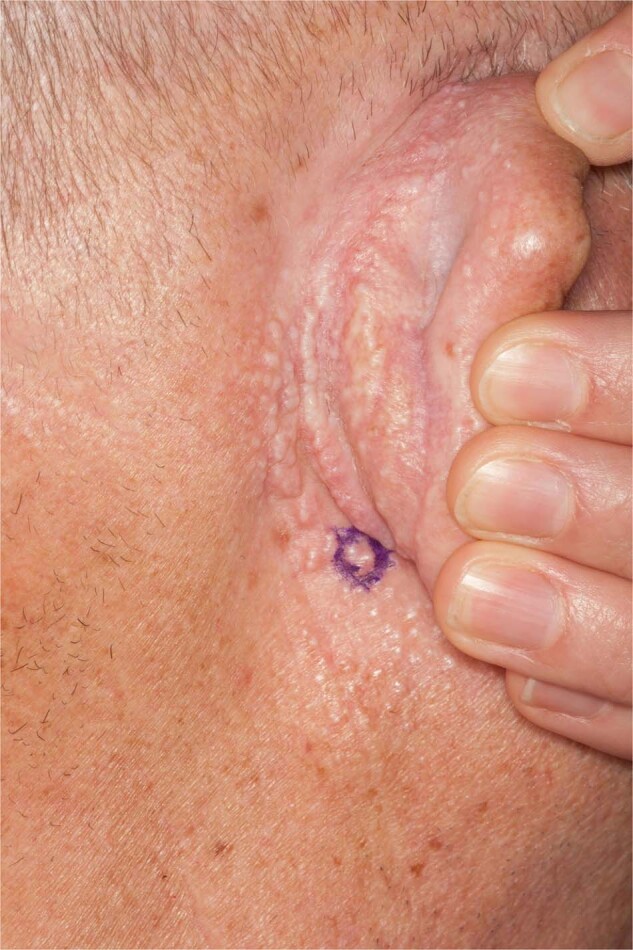
Scleromyxedema. Discrete and coalescing, firm papules of the posterior ear and postauricular region.

Scleromyxedema, a primary dermal diffuse mucinosis, was first described by Dubruilh and Reitmann as a skin disease similar to scleroderma [101, 102]. Mucinoses are characterized by mucin deposits in connective tissue. The skin demonstrates dense, firm, waxy, reddish or skin-colored, dome-shaped or flat-topped papules of 2–3 mm in size. It typically involves the hands, head, upper trunk, and thighs (Figs. 5 and 6). Scleromyxedema can lead to longitudinal furrows in the glabella, also called *facies leonina*. Deep furrowing on the trunk or limbs associated with redundant skin folds is also observed (*Shar-Pei sign*). Few patients also present with a central depression with a raised margin over the proximal interphalangeal joints (*donut sign*), sclerodactyly and Raynaud syndrome. Typically, patients do not have telangiectasia and cutaneous calcinosis in contrast with systemic sclerosis. Major complications of scleromyxedema are contractures of joints and articulations [103].

A monoclonal spike is found in 90% of patients with scleromyxedema, usually IgG lambda. However, IgA kappa, IgA lambda, and IgM kappa monoclonal spikes can also be found. Many neurological complications may arise with scleromyxedema: carpal tunnel syndrome, peripheral sensory and/or motor neuropathy, gait disorder, stroke, seizures, psychosis, and dermato-neuro syndrome [104]. The dermato-neuro syndrome is a flu-like prodrome followed by seizures and coma [105]. Other complications include dysphagia, scleroderma renal crisis-like, arthralgia, inflammatory myopathy, and severe destructive polyarthritis [106, 107]. Cardiovascular manifestations including heart failure with reduced ejection fraction, myocardial ischemia and pericardial may occur [108]. The pathogenesis of cutaneous and extracutaneous manifestations is unknown. Many authors hypothesize that M-protein may stimulates the proliferation of fibroblasts and increase mucin formation [103, 109]. However, there is no correlation between M-protein levels and clinical manifestations.

Diagnostic criteria that should be fulfilled to confirm scleromyxedema [110]:

1. Generalized papular and sclerodermiform eruption
2. Mucin deposits, fibroblast proliferation, and fibrosis on histopathology
3. Monoclonal gammopathy
4. Absence of thyroid disease

Without adequate treatment, scleromyxedema tends to progress, may result in death, and spontaneous regression is exceptional [111]. Two prospective non-randomized trials showed that intravenous immunoglobulins (IVIg) 2 grams/kilogram each month are associated with significant clinical improvement [112, 113]. In a systemic review, Haber & al. showed cutaneous improvement in 69% of patients (*n* = 47) with immunoglobulin as single therapy. In addition, systemic corticosteroids, thalidomide, and autologous stem cell transplantation were respectively associated with 73, 69, and 100% improvements of cutaneous manifestations of scleromyxedema [114]. In context of dermato-neuro syndrome, the combination of IVIg and systemic corticosteroids are the treatment of choice according to European guidelines [115]. Lenalidomide [116] or bortezomib therapy [117–119] may achieve rapid improvement of cutaneous and systemic manifestations of scleromyxedema [120]. However, more studies addressing these issues are warranted.

### Scleredema

**Fig. 7:**
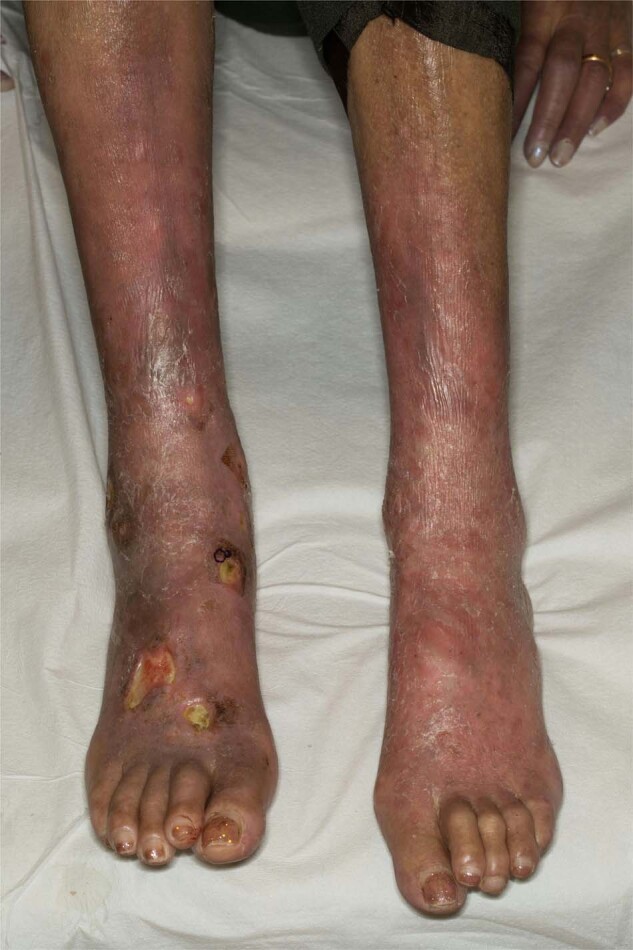
Scleredema. Lower extremity ulcerations associated with woody induration and erythema.

Scleredema, also known as scleredema adultorum of Buschke, is a rare sclerotic skin disease occurring with diabetes mellitus, streptococcal infection, and monoclonal gammopathy. The M-protein is usually an IgG, with predominance of kappa light chain over lambda [121–123]. An excessive dermal mucin and collagen accumulation lead to a symmetrical widespread, thickening, and non-pitting induration of the skin [124]. Some patients also have a *peau d’orange* appearance and ulcers (Fig. 7). The condition tends to be chronic and slowly progressive. The skin lesions generally involve the neck, upper trunk, and upper extremities [125]. The histopathology demonstrates thickened reticular dermis with swelling of collagen bundles. The epidermis is usually normal and there is an absence of fibroblast proliferation in contrast to scleromyxedema. There is a lack of evidenced-based treatment of scleredema. Skin-directed therapy can include ultraviolet (UV) light phototherapy (including narrowband UVB or UVA-1). A case report showed significant improvement with bortezomib and intravenous immunoglobulin [126]. In two other cases report, the combination of cyclophosphamide, bortezomib, and dexamethasone (CyBorD) led to a marked clinical and hematological improvement [127, 128].

### TEMPI syndrome

TEMPI syndrome is defined by telangiectasia (T), erythrocytosis with elevated erythropoietin (E), monoclonal M-protein (M), perinephritic fluid collection (P), and intrapulmonary syndrome (I). This is a rare syndrome, described for the first time in 2011 by Sykes & al in four men and two women [129]. The IgG kappa monoclonal protein predominated. However, cases of IgA lambda, IgG lambda, and IgE lambda are also reported. Usually, patients have less than 30 g/L of M-protein spike [130].

The main skin changes associated with TEMPI syndrome are telangiectasias characterized by persistence of dilated capillary vessels with spider-like appearance or small maculopapular red lesions. Telangiectasias are more prominent on the trunk, face, and upper extremities. Erythrocytosis and extremely elevated serum erythropoietic are present in almost all patients. The JAK2 V617F is absent and allows the exclusion of polycythemia vera. The pathogenesis of TEMPI syndrome is poorly understood. It has been hypothesized that erythrocytosis could be explained by renal damage related to monoclonal immunoglobulin deposition resulting in local hypoxia and elevated EPO levels. Plasma cells might also stimulate bone marrow erythroid progenitor cells and thus exacerbate erythrocytosis [130, 131].

Perinephritic fluid collection is associated with palpable abdominal mass, bilateral flank fullness, and hypertension. However, acute renal injury and proteinuria are uncommon manifestations. Kidney biopsy may demonstrate hypertensive vascular change, light chain deposits, and/or amyloidosis [132, 133]. The presence of lymphangiectasia and interstitial edema is less common [133, 134].

Right-to-left intrapulmonary shunt and hypoxemia (oxygen saturation < 90%) are commonly found in patients with TEMPI syndrome. The degree of shunting must be demonstrated with 99mTc macroaggregated albumin scintigraphy. Echocardiography using saline contrast shows late appearance of left-sided bubbles. Patients have progressive dyspnea on exertion and will ultimately need supplemental oxygen [135].

Treatment with bortezomib-based regimens led to eradication of monoclonal gammopathy and complete resolution of all the symptoms associated with the TEMPI syndrome [133, 136, 137]. Bortezomib-based induction followed by autologous stem cell transplantation with high-dose melphalan might be a viable option [138]. Promising responses have been observed with lenalidomide [139] and daratumumab [140].

### Idiopathic systemic capillary leak syndrome

Idiopathic systemic capillary leak-syndrome (SCLS), also known as Clarkson syndrome, is characterized by generalized edema/anasarca associated with hypovolemia and leakage of intravascular fluid into extravascular space. Less than 260 cases have been reported.

The SCLSC diagnosis criteria are composed of the “3Hs”:

1. Hypotension (systolic blood pressure < 90 mm Hg)
2. Hemoconcentration (hematocrit > 49–50% in men and 43–45% in women)
3. Hypoalbuminemia (albumin < 30 g/L).

Episodes of edema are typically recurrent with a quiescent phase [141]. Between 68 and 85% of SCLS patients will have a persistent monoclonal protein, usually IgG or IgA kappa [142]. Pulmonary edema, acute kidney injury, pleural and pericardial effusion are frequent complications. The initial evaluation includes the exclusion of sepsis, anaphylaxis, hereditary angioedema, and proteinuria. In addition, rapid spontaneous recovery in combination with aggressive fluid resuscitation can worsen hypervolemic state and pulmonary edema. Few patients developed compartment syndrome with rhabdomyolysis necessitating fasciotomies [143].

Treatment of acute SCLC includes conservative fluid resuscitation with crystalloids, albumin, and intravenous vasopressors [144]. Intravenous immune globulin (IVIg) may be used in refractory hypotensive patients [145]. Few studies have shown that terbutaline and theophylline administered orally reduced frequency and severity of SCLC episodes. Increasing cyclic AMP activities might reduce inflammatory signal pathways while attenuating endothelial permeability [146–148]. Few studies described successful prevention of SCLC episodic frequency with prophylactic IVIG 2 grams/kilogram every month tapered to 1 gram/kilogram every month after achieving remission [149, 150]. No data support the combination of IVIg and terbutaline or theophylline, but this can still be effective.

### Acquired Cutis laxa

Acquired cutis laxa is a connective tissue disorder resulting in loose, wrinkled, and redundant skin due to inelastic skin. It can be associated with MGUS, myeloma, lymphoproliferative syndrome, and heavy chain deposition disease [21, 151].

**Fig. 8:**
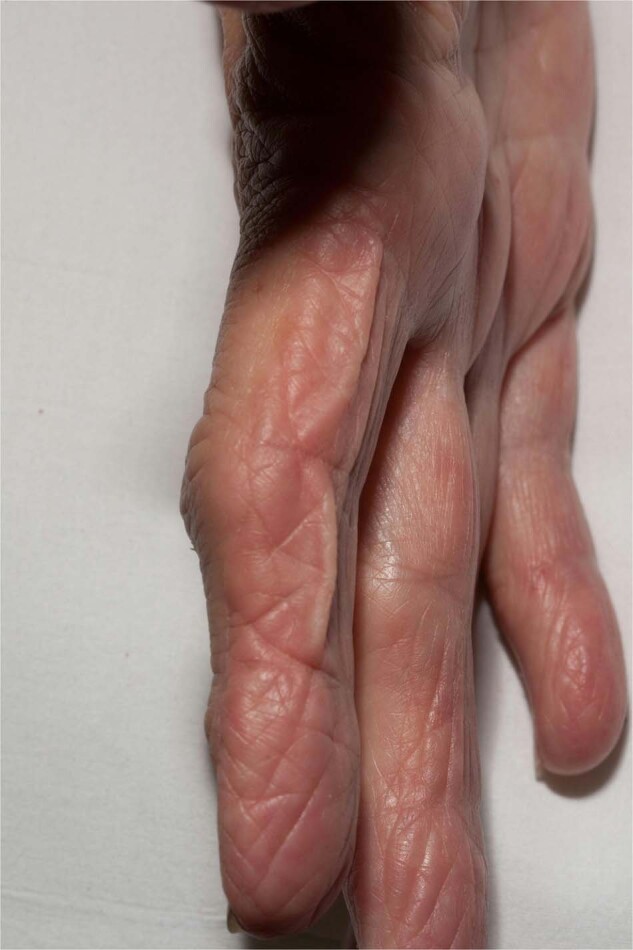
Acquired cutis laxa. Laxity of the skin involving the fingers.

Deposition of immune complexes cause release of inflammatory cytokines which destroy the elastic fibers. Excess of light chains may also alter elastin production by activation of the alternative complement pathway. However, the presence or not of immunoglobulin deposition on elastin fibers in unclear [152, 153]. The generalized form is characterized by *hound dog* facies following lax skin of the trunk and extremities (Fig. 8). Some localized forms also exists, including distal extremity involvement associated with systemic amyloidosis or multiple myeloma [154]. Very few studies describe stabilization or amelioration of cutis laxa following treatment of the underlying monoclonal gammopathy. Early management with plastic surgery with reconstructive procedures is usually required [155].

### Pyoderma gangrenosum

Pyoderma gangrenosum (PG) is a neutrophilic dermatosis that presents as solitary or multiple lesions. The first description of PG was done by Brunsting, Goeckerman, and O’Leary in 1930 [156]. They believed that a skin infection was the cause of the PG. However, this was a misnomer since PG is not a skin infection nor a classic gangrenous condition. PG can be associated with trauma, inflammatory bowel disease, inflammatory arthritis, hematological malignancy, MGUS, and solid malignancy [157, 158]. There is a predominance of IgA gammopathy in patients with monoclonal gammopathy and PG [159].

There are six major clinical variants of pyoderma gangrenosum: ulcerative, bullous, pustular, vegetative, peristomal, and postoperative. The ulcerative PG is the most frequent variant associated with monoclonal gammopathy [160]. PG is generally characterized initially by a pustule with or without peripheral erythema and/or reddish-violaceous appearance. The initial lesion can also be an inflammatory nodule or pustule and it tends to rapidly evolve to erosion and/or necrotic ulcers with violaceous borders and undermined edges [161]. Diagnostic criteria require exclusion of other diagnoses including infection (Table 4) [162].

**Table 4:** Pyoderma gangrenosum diagnostic criteria.

Major criteria	• Biopsy of ulcer edge demonstrating a neutrophilic infiltrate
Minor criteria	• Exclusion of infection • Pathergy • Personal history of inflammatory bowel disease or inflammatory arthritis • History of papules, pustules, or vesicles that rapidly ulcerated • Peripheral erythema, undermining border, and tenderness at site of ulceration • Multiple ulceration (at least one on an anterior lower leg) • Cribriform or wrinkled paper scar at site of healed ulcers • Decrease in ulcer size within one month of initiating immunosuppressive medication

1 major criteria and at least 4 minor criteria must be fulfilled. Adapted from ref. ^162^.

Achieving remission of the primary hematological malignancy does not always lead to resolution of PG. Combination of systemic and topical corticosteroids is the first line therapy [163]. Cyclosporine is used as an alternative first-line therapy in cases where corticosteroids are contraindicated or as second-line treatment in patients whose disease did not respond to corticosteroids [158, 164]. Biological agents, like infliximab, show promising efficacy with PG. However, their use should be restricted in patients with monoclonal gammopathy since biological agents may promote flare-up of M-protein [165, 166]. Mycophenolate mofetil, cyclophosphamide, danazol, IL-1 inhibitors (anakinra and canakinumab), and azathioprine are options for patients with refractory PG [167–169].

### Sweet syndrome

Sweet syndrome (acute febrile neutrophilic dermatosis) is an inflammatory condition associated with autoimmune disease, infections, hematologic malignancies, and solid malignancies. Lymphoma, acute myeloid leukemia, myelodysplastic syndrome, chronic myeloid leukemia, and hairy cell leukemia are some possible hematologic etiologies. Studies also reported MGUS and multiple myeloma as etiologies of Sweet syndrome [170–176].

The cutaneous lesions are usually tender, edematous, and erythematous plaques with a symmetric distribution. The lesions are typically localized to the face, trunk, neck, and upper extremities. In patients with malignancies, the lesions may be ulcerated and vesicular (at times localizing to the dorsal hands), or characterized by flaccid bullae and mimic the morphology of pyoderma gangrenosum (bullous variant) [177]. Less common presentations include erythematous nodules (subcutaneous Sweet syndrome) and pustular lesions of the dorsal hands [178, 179].

The major diagnostic criteria of classical Sweet syndrome are shown in Table 5 [180, 181]. Histopathology shows infiltration of neutrophils in the dermis and subcutaneous fat with endothelial swelling, and prominent edema in the superficial dermis. There is an absence of leukocytoclastic vasculitis. First-line treatments are high-potency topical or systemic corticosteroids. Potassium iodide, dapsone, and colchicine may also be used due to their anti-neutrophilic activity [182].

**Table 5:** Sweet syndrome diagnostic classification criteria.

Major criteria	1. Abrupt onset of painful erythematous plaques or nodules 2. Histopathologic evidence of a dense neutrophilic infiltrate without evidence of leukocytoclastic vasculitis
Minor criteria	1. Fever > 38 °C 2. Associated with inflammatory disease or pregnancy, or preceded by upper respiratory infection, or gastro-intestinal infection, or vaccination 3. Excellent response to treatment with systemic corticosteroid or potassium iodine 4. Abnormal laboratory results at presentation a. Eythrocyte sedimentation rate > 20 mm/h b. Positive C-reactive protein c. >8000 leucocytes d. >70% neutrophils

Both major criteria and at least 2 minor criteria must be fulfilled. Adapted from ref. ^181^.

### Erythema elevatum diutinum (EED)

**Fig. 9:**
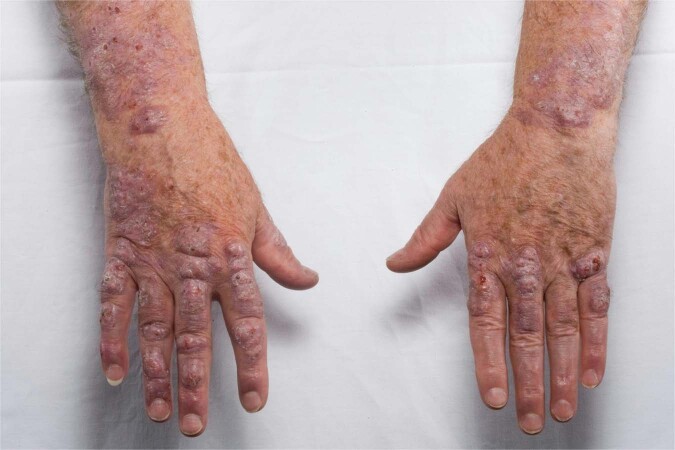
Erythema elevatum diutinum. Coalescing pink-to-violaceous plaques and nodules of the dorsal hands and wrists (including over extensor joints).

EED is a rare chronic dermatosis. The skin lesions are described as red-brown, violaceous or yellow papules, plaques, or nodules (Fig. 9). EED mostly involves the extensor surfaces of joints, axilla, posterior auricular area, buttocks, soles, larynx, and acral sites. Atypical presentations as annular lesions or verrucous plaques are also seen [183–188].

EED is associated with monoclonal gammopathy, more frequently IgA MGUS [185] or multiple myeloma [189, 190]. EED is also found with HIV infection [191, 192], tuberculosis [193], hepatitis B infection [192], myelodysplastic syndrome [194], lymphoma [195], and autoimmune disease [190].

Some patients with EED will have extracutaneous manifestations, including arthralgia, scleritis, panuveitis, ulcerative keratitis, neuropathy, and ulceration. On histopathology, early lesions demonstrate neutrophilic infiltration and typical leukocytoclastic vasculitis in the upper dermis to the mid-dermis [190]. With the progression of the disease, the papillary and periadnexal dermis become involved. Granulation tissue, xanthomatization, and storiform fibrosis are also seen [185, 196, 197]. Dapsone is the preferred first-line treatment and is associated with improvement of cutaneous and extracutaneous symptoms (although late nodular cutaneous lesions may be more recalcitrant to treatment).

### Subcorneal pustular dermatosis

Subcorneal pustular dermatosis (SPD) is a neutrophilic dermatosis characterized by a relapsing asymptomatic eruption of superficial pustules occurring predominantly in intertriginous sites. SPD involves the trunk, axillae, groin, abdomen and flexural aspect of the proximal extremities. The pustules normally measure a few millimeters in diameter, but can be larger and become flaccid bullae, called “half-half” blisters (with clear fluid on the superior portion and pus on the inferior portion, also known as “hypopyon”) [198].

SPD frequently is a chronic benign disease. However, SPD is also found in patients with monoclonal gammopathy, usually IgA gammopathy [199–202]. The treatment of choice for SPD is dapsone [203].

### Perspective on cutaneous manifestations of gammopathy

Cutaneous manifestations of monoclonal gammopathy are an oftentimes overlooked manifestation of clonal proliferative dyscrasia. These cutaneous manifestations are associated with heterogeneous clinical manifestations and prognosis (Table 6). The treatment depends mainly on the severity of the cutaneous disease (Table 7). We sought to clarify the classification and cutaneous manifestations of monoclonal gammopathy, while extending the concept of monoclonal gammopathy of clinical significance. Additional studies are needed to investigate the pathogenesis of monoclonal gammopathy-associated skin disease and therapy options.

**Table 6:** Summary of Group II cutaneous manifestations associated with monoclonal gammopathy.

Entity	Criteria	Skin findings	Other signs
POEMS*	Mandatory criteria 1. Polyneuropathy (typically demyelinating) 2. Monoclonal plasma cell-proliferative disorder Major criteria 3. Castleman disease 4. Sclerotic bone lesion 5. VEGF elevation Minor criteria 6. Organomegaly 7. Extravascular volume overload 8. Endocrinopathy 9. Skin change 10. Papilledema 11. Thrombocytosis/polycythemia	Hyperpigmentation, hypertrichosis, glomeruloid hemangiomas, plethora, lipodystrophy, acrocyanosis, flushing, white nails	Clubbing, weight loss, pulmonary hypertension, restrictive pulmonary syndrome, diarrhea, thrombotic disease, hyperhidrosis
Schnitzler syndrome	Obligate criteria 1. Chronic urticarial rash 2. Monoclonal IgM or IgG Minor criteria 3. Recurrent fever 4. Abnormal bone remodeling with or without bone pain 5. Neutrophilic urticarial dermatosis on skin biopsy 6. Leukocytosis and/or elevated CRP	• Urticarial recurrent rash, mostly on the trunk and limb • Rose or red macules or raised papules or plaques • Triggered by stress or physical exercise • Dermographism	• Fatigue • Pain in joint, muscle, and/or bone • Enlarged lymph nodes • Organomegaly (liver or spleen)
Necrobiotic xanthogranuloma	Major criteria 1. Cutaneous papules, plaques, and/or nodules, most often yellow or orange 2. Palisading granulomas with lymphoplasmacytic infiltrate and zones of necrobiosis on skin biopsy 3. Periorbital distribution of cutaneous lesions 4. Paraproteinemia, often IgG-lambda, plasma cell dyscrasia or lymphoproliferative disorder	• Periorbital skin is the most common site • Yellow-orange, reddish-brown or violaceous indurated papules and nodules that gradually enlarge to form infiltrative plaques • Central aspect of larger plaques can be atrophic with prominent telangiectasia • Scarring and ulceration	• Ocular involvement • Proptosis • Limited extraocular mobility • Blurred vision, dry eyes, diplopia • Acute transient vision loss
Scleromyxedema	1. Generalized papular and sclerodermoid eruption 2. Microscopic triad associating dermal mucin deposition, thickened collagen, and fibroblast proliferation or an interstitial granuloma annulare-like pattern 3. Evidence of monoclonal gammopathy 4. Absence of thyroid disease	• Firm, waxy, dome-shaped or flat-topped papules • Shiny and indurated appearance of skin • Involve hands, head, neck, upper trunk, and thighs • Glabella typically involved • Shar-Pei sign • Pruritus uncommon	• Peripheral sensory and motor neuropathy • Carpal tunnel syndrome • Dermato-neuro syndrome • Dysphagia • Scleroderma-like renal crisis
Tempi syndrome	Major Criteria 1. Telangiectasias 2. Elevated serum erythropoietin and erythrocytosis 3. Monoclonal gammopathy 4. Perinephric fluid Minor criteria 5. Intra-pulmonary shunting 6. Others: venous thrombosis	• Telangiectasias most prominently on the face, hands, upper back, and chest	• Lack of JAK2 mutation • Normal VEGF levels
Idiopathic systemic capillary leak-syndrome	1. Intravascular hypovolemia 2. Generalized edema 3. Diagnosis triad: Hypotension, hemoconcentration, and hypoalbuminemia 4. Diagnostic of exclusion	• Generalized edema/anasarca associated with hypovolemia and hypoalbuminemia • Spontaneous periodic edema	• Acute renal failure • Pulmonary edema • Pericardial or pleural effusion • Compartment syndrome

*POEMS syndrome is a Group I skin disease monoclonal gammopathy.

**Table 7:** Treatments of cutaneous manifestations associated with monoclonal gammopathy.

POEMS	• If 1–2 bone lesions and negative bone marrow: radiation • If > 2 bone lesion or positive bone marrow: lenalidomide/dexamethasone followed by ASCT
Scleromyxedema	First line • High-dose immunoglobulin Second line • Thalidomide or lenalidomide • Systemic corticosteroid Third line • Bortezomib • ASCT • Melphalan
Schnitzler syndrome	First line • IL-1 inhibitor (anakinra, canakinumab, rilonacept) Second line • Waldenström macroglobulinemia therapy
Necrobiotic xanthogranuloma	First line • Melphalan • Chlorambucil Second line • IVIg • Corticosteroid
Scleredema	First line • IVIg and corticosteroid Second line • Bortezomib-based regimen • Ultraviolet (UV) light phototherapy
Tempi syndrome	• Bortezomib-based regimen followed by ASCT with high-dose melphalan
Idiopathic systemic capillary leak-syndrome	Acute episode • Fluid resuscitation • Intravenous vasopressor • IVIg Prophylaxis • Theophylline or terbutaline • IVIg
Acquired cutis laxa	• Plastic surgery with reconstructive procedures
Pyoderma gangrenosum	First line • Systemic corticosteroid • Cyclosporine Second line • Mycophenolate mofetil • Azathioprine Third line • Cyclophosphamide • Intravenous immunoglobulin • Chlorambucil
Sweet syndrome	• Intralesional or topical corticosteroids • Systemic corticosteroid • Refractory cases: dapsone, potassium iodide or colchicine
Erythema elevatum diutinum	• Dapsone
Subcorneal pustular dermatosis	• Dapsone

*ASCT* autologous stem cell transplantation, *IVIg* Intravenous immunoglobulin
